# Cord blood procalcitonin and soluble urokinase-type plasminogen activator receptor in predicting histological chorioamnionitis – A pilot study

**DOI:** 10.1016/j.eurox.2026.100475

**Published:** 2026-07-24

**Authors:** Kati Jalkanen, Outi Tammela, Anita Virtanen, Janne Aittoniemi, Heini Flinck, Sinikka Ampuja, Heini Huhtala, Kati Tihtonen

**Affiliations:** a Department of Obstetrics and Gynecology, Tampere University Hospital, Wellbeing Services County of Pirkanmaa, Tampere, Finland; b Department of Pediatrics, Tampere University Hospital, Wellbeing Services County of Pirkanmaa, Tampere, Finland; c Department of Clinical Microbiology, Fimlab Laboratories, Tampere University Hospital, Tampere, Finland; d Department of Pathology, Tampere University Hospital, Tampere, Finland; e Faculty of Social Sciences, Tampere University, Tampere, Finland; f Faculty of Medicine and Health Technology, Tampere University, Tampere, Finland

**Keywords:** Procalcitonin, Soluble urokinase-type plasminogen activator receptor, Cord blood, Amniotic fluid, Histological chorioamnionitis, Fetal inflammatory response syndrome

## Abstract

**Aims:**

Chorioamnionitis, caused by microbial invasion of amniotic cavity or sterile inflammation, can lead to prematurity and cytokine storm of fetus known as fetal inflammatory response syndrome (FIRS). Histological chorioamnionitis (HCA) on fetal side of placenta is a surrogate to FIRS. Cord blood (CB) reflects intrauterine inflammation. Our aim was to evaluate if more novel biomarkers procalcitonin (PCT) and soluble urokinase- type Plasminogen Activator Receptor (suPAR) were useful for identifying fetuses/neonates with high risk for FIRS/HCA.

**Methods:**

Twenty two women with a clinically confirmed preterm premature rupture of membranes (PPROM) or a suspicion of chorioamnionitis without PPROM (uterine tenderness, maternal fever ≥38°C, leukocytosis, fetal tachycardia ≥160 beats/minute) were enrolled at gestational weeks 23^+0^–34^+6^. Interleukin-6 (IL-6), PCT and suPAR were measured in cord blood samples obtained immediately after birth. The fetal side HCA was used as a surrogate for FIRS.

**Results:**

The cord blood concentration of IL-6 was significantly higher (p = 0.01) and had the best predictability for fetal side HCA (AUC 0.96, p = 0.03). Cord blood concentrations of PCT tended to be higher (p = 0.05), and to show significance for predicting fetal-side HCA (AUC 0.91, p = 0.07). No significant findings were found for suPAR.

**Conclusion:**

Cord blood IL-6 had the best ability to predict fetal side HCA. Cord blood PCT may be a promising biomarker. There were no significant findings for cord blood suPAR, suggesting its limited value as a biomarker in this context.

## Introduction

One-third of preterm deliveries are caused by preterm premature rupture of membranes (PPROM). PPROM is a significant risk factor for various adverse neonatal outcomes such as prematurity, respiratory distress, and neonatal sepsis. Delivery is timed to find the optimal balance between the risks of prematurity and the risk of chorioamnionitis with advancing gestational age. Chorioamnionitis may occur with clinical symptoms or may be rather asymptomatic. Fetal inflammatory response syndrome (FIRS), a pathological fetal systemic inflammation and a consequence of intra-amniotic infection (IAI) or sterile inflammation, can worsen neonatal outcomes [1], [2], [3]. Inflammation in different fetal organs, especially in the brain and lungs, is associated with long-term neurological and respiratory morbidities. When such inflammation takes the form of FIRS, it is also associated with early-onset sepsis (EOS), for which the administration antibiotics soon after onset is essential [4], [5], [6]. It would be beneficial if neonates with high risk of FIRS could be detected by using cord blood (CB) sample especially in cases without clinical symptoms of chorioamnionitis.

Interleukin- 6 (IL-6) is a pro-inflammatory cytokine. Elevated amniotic fluid (AF) IL-6 correlates with fetal inflammation/infection. High plasma IL-6 concentration from CB-derived samples as well as neonatal samples has been used as a hallmark of FIRS; however, further investigation in neonates in clinical practice is warranted [1], [2], [3], [4].

Procalcitonin (PCT) and soluble urokinase-type plasminogen activator receptor (suPAR) are novel inflammatory markers. PCT, a pro-hormone of calcitonin, is involved in calcium homeostasis, and it increases in response to pro-inflammatory stimulus, especially of bacterial origin. It increases faster than C-reactive protein (CRP) in response to severe bacterial infections in adult patients [7]. In term and preterm neonates, PCT can be used as a valuable diagnostic tool for suspected EOS and late-onset sepsis (LOS) as well as to guide antibiotic treatment [8], [9]. Furthermore, PCT concentration is not affected by corticosteroid medication, which is widely used to promote maturation of the fetal lung in pregnancies with a risk of preterm delivery [10].

SuPAR is a biomarker for sepsis, serious infections, and organ failures in adult patients, and neutrophils are the main source of circulating suPAR in systemic inflammation [11], [12]. Chorioamnionitis can be restricted to the maternal side or extended to the fetal side of the placenta, including the umbilical cord (funisitis). In chorioamnionitis involving the umbilical cord in the form of funicitis, fetal neutrophils are found within the umbilical vessels, which is a histological feature corresponding to FIRS. Histological fetal side chorioamnionitis can be used as a proxy of FIRS [1], [2], [3].

We hypothesized that CB concentrations of more recently established inflammatory biomarkers mostly used in adult patients—PCT and suPAR—along with the conventional inflammatory marker IL-6 would be higher in the case of histological fetal side chorioamnionitis following pregnancies complicated by PPROM. To test this hypothesis we compared the CB concentrations of inflammatory markers between pregnancies with histological fetal side and without histological fetal side chorioamnionitis. We also evaluated the predictability of the inflammatory markers for histological fetal side chorioamnionitis and how the inflammatory markers from the CB samples correlate with the AF samples, which are mostly used to assess intrauterine infection during pregnancy.

## Material and methods

This prospective pilot study was performed between 2015 and 2017 at Tampere University Hospital (TAUH), Finland. All patients provided a written informed consent of the study. The study was approved by the TAUH Ethical Committee (Ethical code R15008) and was conducted according to the principles of the Declaration of Helsinki.

The inclusion criteria were pregnant women from gestational week 23^+0^ to 34^+6^ with a PPROM diagnosis or symptoms/suspicion of IAI without PPROM. Gestational age was calculated according to the last menstrual period and corrected on the basis of first-trimester ultrasonographic screening results if needed. PPROM was diagnosed by a sterile speculum examination confirming pooling of amnion fluid and, if needed, using Actim Prom® (Medix Biochemica, Kauniainen, Finland). Suspicion of IAI without PPROM was based on the presence of at least two of the following symptoms: uterine tenderness, maternal fever ≥ 38 °C, fetal tachycardia ≥ 160 beats/minute, maternal leukocytosis, or elevated CRP. The exclusion criteria were gestational weeks under 23^+0^ or above 34^+6^, fetal anomaly, and multifetal pregnancy.

On admission, all the women were tested by vaginal swab culture for *Streptococcus agalactiae*. Urine bacterial culture was obtained. Chlamydia and Neisseria gonorrhoeae were tested from urine samples using polymerase chain reaction (PCR).

All the patients were managed according to the local clinical guidelines and admitted to a prenatal ward. Antibiotics (cefuroxime 1.5 g × 3 intravenously and azithromycin 500 mg × 1 per os) were routinely administered for 72 h. Corticosteroids (Celestone® 12 mg twice 24 h apart) were given, and during this time, tocolytic therapy was also administrated if needed. Fetal cardiotocography (CTG) was recorded daily.

Ultrasound guided transabdominal amniocentesis was performed on the prenatal ward by a senior obstetrical consultant and repeated weekly until delivery. AF glucose, lactate dehydrogenase, and bacterial PCR for clinical use were analyzed. AF samples not used for clinical purposes were centrifuged and stored at −70 ◦C until analyzed. Results considering AF and maternal plasma suPAR, PCT, and IL-6 have been published separately [13]. AF and CB plasma suPAR, PCT and IL-6 were analyzed. SuPAR level was determined using suPARnostic® AUTO Flex ELISA (ViroGates, Birkerød, Denmark), PCT level using LIAISON® BRAHMS PCT® II GEN (DiaSorin, Saluggia, Italy), and IL- 6 level using HS ELISA or Quantakine® ELISA (R&D Systems, Minneapoli, MN,US) assay according to the manufacturer`s instructions.

If there was no clinical symptoms of chorioamnionitis, signs of fetal distress or evidence for IAI based on the AF sample (AF glucose ≤ 1 mmol/l and LD ≥ 280 U/l cutoffs were used), pregnancy was continued. Otherwise, the decision of delivery was made.

**Fig. 1:**
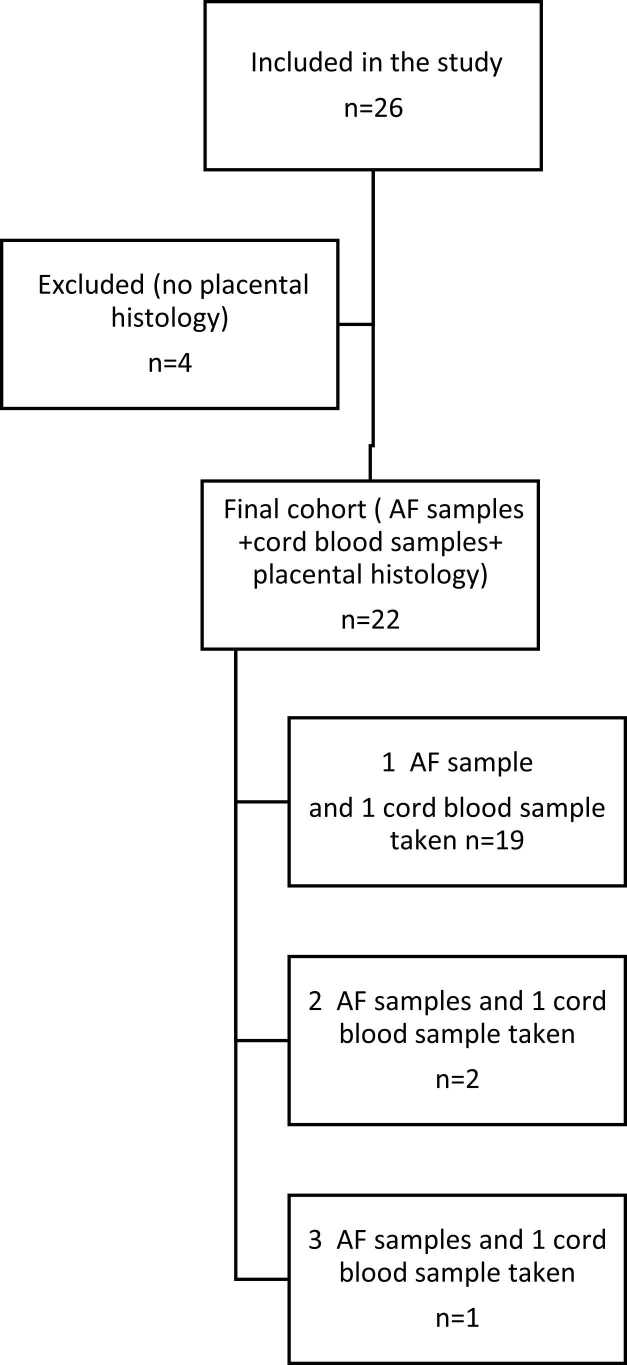
The patient flow diagram. The final cohort was formed from the AF samples taken closest to delivery.

Overall, 26 women were included in the study, and 31 AF samples were taken. After excluding cases without placental histopathology, 22 women were finally included in the study. For those undergoing amniocentesis more than once, the AF sample closest to delivery were considered for the analysis. Umbilical cord blood samples for suPAR, PCT, and IL-6 were collected after the routine CB gas analysis and thyreotropin analysis and were centrifuged and stored at −70 °C until analyzed. Clinical and laboratory data on women and newborns were collected from hospital medical records. Flowchart of selection process is presented in Fig. 1 (Fig. 1).

Histopathological evaluation was performed by a placental pathologist. Histological chorioamnionitis (HCA) on the maternal or fetal side was estimated using Redline classification and Amsterdam international consensus for placental lesions [14]. Women and neonates with histological fetal side chorioamnionitis are referred to as the HCA group and those with only maternal side chorioamnionitis or without chorioamnionitis as the non-HCA group.

The data was analyzed using IBM SPSS version 28. The data are expressed as median and interquartale range or frequency (n) and percentage (%). We compared continuous variables using the Mann–Whitney *U* test and categorial variables using Fisher’s exact test or Chi-square test. The correlations were calculated using Spearman’s correlation coefficients. Sensitivity and specificity were also calculated. Receiver operating characteristic curves were derived to evaluate the diagnostic performances of AF and CB biomarkers in the prediction of fetal side HCA, and the area under the curve (AUC) and 95% confidence interval were determined. The significance level was set at p value less than 0.05.

## Results

### Study population

Baseline characteristics of the study population are presented in Table 1. The median gestational age at delivery was 30^+4^ (interquartile range [IQR] 25^+0^–40^+3^), and the median interval from entry into the study until delivery was 3 weeks (range 0–98 days). 50% of the deliveries were induced due to suspicion of IAI. The rates of emergency (due to non-reassuring CTG) and elective (due to fetal breech presentation and maternal request) cesarean sections (CSs) were 18% each. The median birthweight was 1380 g (IQR 630–3265 g), and the median hospital stay in the neonatal intensive care unit (NICU) was 23 days (range 0–105 days). None of the neonates had EOS or intraventricular hemorrhage gradus III-IV.Two neonates had necrotizing enterocolitis (NEC) and were treated with antibiotics. Six neonates were diagnosed with respiratory distress syndrome (RDS), bronchopulmonary dysplasia (BPD), or both (HCA group, n = 3; non-HCA group, n = 3).

**Table 1:** Baseline characteristics of parturients and neonates.

	n = 22
Age, years (mean, SD)	30(5)
BMI, kg/m^2^ (median, IQR)	26 (18−45)
Primiparas, %	54.5
Antenatal steroid treatment,%	100
Antenatal antibiotic treatment,%	100
Positive AF PCR,%	22,7
Induction of labor, %	50
GA at PPROM/suspected IAI (median, IQR)	27 + ^4^ (23 +^0-^34 +^5^)
GA at delivery (median, IQR)	30 + ^4^ (25 +^0^−40 +^3^)
Mode of delivery	
vaginal delivery, %	64
crash or emergency cesarean section, %	18
elective cesarean section, %	18
Birth weight, g (median, IQR)	1380 (630–3265)
Days in the NICU (median, range)	23 (0−105)
1 min Apgar (median, IQR)	8 (8−9)
5 min Apgar (median, IQR)	7(7−9)
Arterial cord blood pH(median, IQR)	7.31(7.26–7.40)
Early onset sepsis,%	0
Composite neonatal morbity (RDS,BPD,NEC,IVH gr III-IV),%	36

SD, standard deviation, BMI, body mass index, IQR, interquartile range,

PCR, polymerase chain reaction, GA, gestational age.

PPROM, preterm premature rupture of membranes, IAI, intra-amniotic infection,

NICU, neonatal intensive care unit, RDS respiratory distress syndrome; BPD bronchopulmonary dysplasia; NEC necrotizing enterocolitis; IVH intraventricular hemorrhage.

### Biochemical markers of HCA in CB

**Fig. 2:**
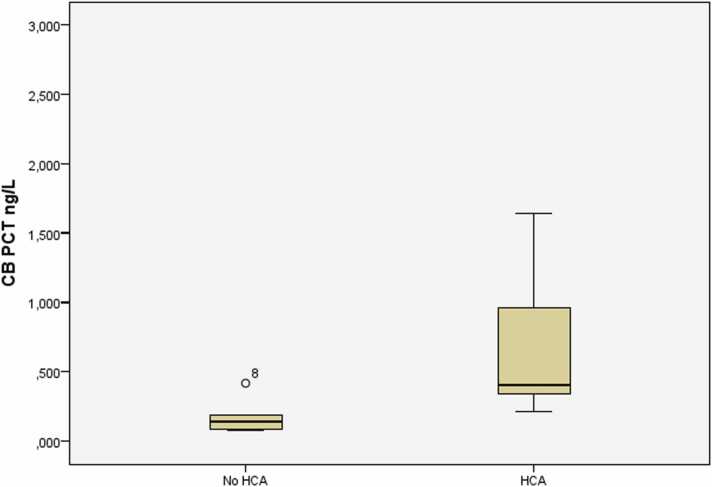
Cord blood PCT levels. No HCA(n = 9), HCA(n = 13). The median concentrations of CB PCT were lower in those without HCA than those with HCA; 138 ng/L(range 0,087−0,245 ng/L) vs 0,406 ng/L (range 0,342−1390 ng/L), p = 0,05. HCA; histological chorioamnionitis.

**Fig. 3:**
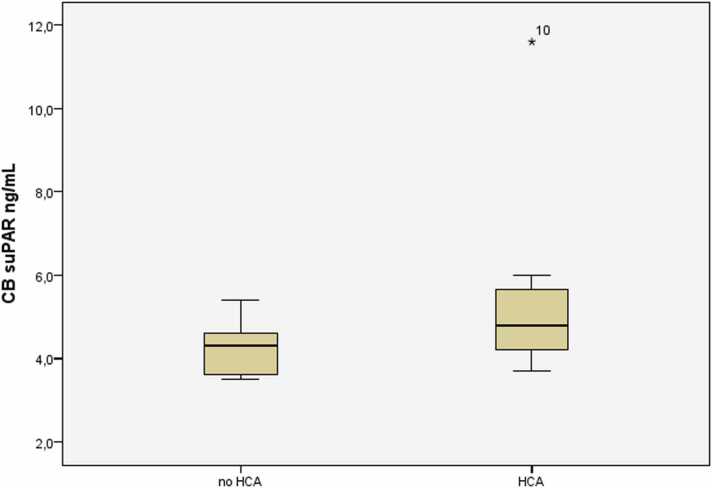
Cord blood suPAR levels. No HCA (n = 9), HCA (n = 13). The median concentrations of CB suPAR were lower in those without HCA than those with HCA;4,3 ng/mL (range 3,5−4,8 ng/mL) vs 4,8 ng/mL(range 4,1−5,9 ng/mL), p = 0,180. HCA; histological chorioamnionitis.

HCA was diagnosed in 59% (13/22) and non-HCA in 41% (9/22) of pregnancies. The concentrations of the inflammatory markers in the HCA and non-HCA groups are presented in Table 2. The median CB IL-6 was significantly higher (968 ng/L vs. 13 ng/L, p = 0.01) and CB PCT tended to be higher (0.4 ng/L vs 0.1 ng/L, p = 0.05) in the HCA group than in the non-HCA group (Fig. 2). No significant difference was found in suPAR concentrations between the groups (Fig. 3).

**Table 2:** Biochemical markers in cord blood and in amniotic fluid.

	HCA n = 13 Median (IQR)	no HCA n = 9 Median (IQR)	p-value
Biochemical markers in cord blood			
SuPAR (ng/mL)	4.80(4.10–5.90)	4.3 (3.50–4.80)	0.180
PCT (ng/L)	0.406 (0.342–1.390)	0.138 (0.087–0.245)	0.05
IL-6 (ng/L)	968.0 (100.0–1689.0)	12.99 (5.74– 60.80)	0.01
Biochemical markers in amniotic fluid			
SuPAR (ng/mL)	58.0 (13.2–204.0)	28.5 (12.4–59.5)	0.071
PCT (ng/L)	0.069 (0.027–0.156)	0.106 (0.035–0.214)	0.209
IL-6 (ng/L)	10000 (455–19600)	1360 (81–10800)	0.110

IQR, interquartile range, SuPAR, soluble urokinase-type plasminogen activator receptor, PCT, procalcitonin, IL-6, interleukin 6.

### Predictive performance of biochemical markers in CB

The predictive performance of the biomarkers is presented in Table 3. In the CB samples, IL-6 was the most accurate biomarker to identify HCA, with 100% sensitivity, 83% specificity (cutoff value 25.10 ng/mL), and 0.96 AUC (p = 0.03). CB PCT also demonstrated a high sensitivity of 91%, with a comparable specificity of 83%, when a 0.23 ng/mL cutoff value was used. PCT AUC for HCA was 0.91 (p = 0.07) which showed a tendency for significance.SuPAR had the poorest predictability: 73% sensitivity, 50% specificity (cutoff value 4.25 ng/mL), and 0.71 AUC (p = 0.2).

**Table 3:** Area under the curve from ROC-analysis, sensitivity and specificity of biomarkers in amniotic fluid (AF) and cord blood (CB) for histological fetal infection.

Biochemical marker	AUC	95% CI	p-value	cut-off	sensitivity	specificity
AF SuPAR (ng/mL)	0.71	0.45–0.97	0.193	32.50	69	67
AF PCT (ng/L)	0.35	0.07–0.62	0.336	0.087	46	44
AF IL-6 (ng/L)	0.71	0.45–0.97	0.193	3335	69	67
AF Glucose (mmol/L)	0.82	0.63–1.00	0.013	0.550	62	100
AF LD (IU/L)	0.67	0.42– 0.91	0.210	334.50	69	78
CB SuPAR(ng/mL)	0.71	0.45–0.97	0.159	4.25	73	50
CB PCT (ng/mL)	0.91	0.73–1.00	0.07	0.231	91	83
CB IL-6(ng/mL)	0.96	0.85–1.00	0.03	25.10	100	83

AUC, area under the curve, CI, confidential interval.

### Correlations of biochemical markers in CB and AF

In the entire study population, a strong correlation between CB IL-6 and CB PCT (r = 0.878, p < 0.001) was observed, and CB IL-6, PCT, and suPAR tended to have a negative correlation with neonatal birthweight (r = 0.553; 0.561; 0.436 and p = 0.016; 0.017; 0.07, respectively). Furthermore, no significant correlations were found between the CB and AF samples: CB IL-6 and AF IL-6 (r = 0.27, p = 0.274), CB PCT and AF PCT (r = 0.203, p = 0.418), and CB suPAR and AF suPAR (r = 0.393, p = 0.107). However, in the HCA group, CB suPAR and AF suPAR had a significant positive correlation (r = 0.633, p = 0.036).

## Discussion

In this study, conventional inflammatory marker CB IL-6 had the best predictability for HCA compared to recently identified biomarkers—PCT and suPAR. However, there was a tendency for PCT concentrations to be higher in the HCA group and it had very good accuracy in predicting HCA. In addition, there was a strong correlation between CB IL-6 and CB PCT. The findings considering suPAR were insignificant. Lastly, no significant correlations between CB and AF concentrations of IL-6, PCT, and suPAR were found.

IL-6 is a pro-inflammatory cytokine secreted by macrophages in response to pathogens. It stimulates acute phase protein synthesis and the production of neutrophils. High levels of IL-6 in CB are considered diagnostic for FIRS and predictive for EOS [3]. Furthermore, preterm neonatal pro-inflammatory response, including umbilical CB IL-6 levels, is reportedly comparable to term neonatal response [15]. Our findings of significantly higher CB concentrations and the best predictive ability of IL-6 for HCA are in concordance with previous findings [1], [2], [3]. In the present study, high CB IL-6 probably indicates the general active inflammatory response of the fetus with HCA, since the neonates had a low incidence of specific short-term complications associated with fetal infection or inflammation and high IL-6 concentrations. This could be attributed to prophylactic antibiotics use and active delivery when complications were suspected during the pregnancy to avoid neonatal sepsis [16]. Fetal infection/inflammation after a PPROM during pregnancy can be predicted by analyzing the markers in AF. Previous studies found that high AF IL-6 is related to FIRS [3], [17]. In our study, no significant differences in AF IL-6 concentrations were found between the HCA and non-HCA groups. AF IL-6 and CB IL-6 had no significant correlation. Furthermore, while IL-6 could predict HCA in CB, it could not in AF.

PCT is mostly formed by thyroid C cells in healthy people. Inflammation makes liver macrophages, monocytes, and lymphocytes in the lungs and kidneys to form PCT. Previous studies on PCT in neonatal EOS and LOS have been reported. It is speculated that in preterm neonates, the rise of PCT levels is longer and higher because of the immaturity of the immune system and delayed kinetics [8], [18], [19]. We observed a tendency for higher CB PCT in the HCA group even when there were no EOS cases. Perhaps this may be because CB PCT indicates a general immune response in HCA, similar to IL-6. In addition, it is possible that IAI/inflammation with atypical pathogens, such as *Ureaplasma urealyticum*, causes a milder immune response [20]. In our study population, we had five PCR positive AF samples in HCA group, four of them *Ureaplasma* spp and all had a negative blood culture. CB PCT is also known to increase with obstetrical complications, such as fetal asphyxia; however, since none of the neonates in our study had asphyxia, it can be assumed that the increased CB PCT was produced by fetal tissue in response to IAI [9], [21]. CB PCT could be a promising marker for increased risk for FIRS after PPROM. Similarly, as in the case of IL-6, PCT in AF could not predict HCA nor any correlation between AF and CB PCT was found.

SuPAR is a soluble form of the uPAR. UPAR is present in the endothelium and various immune cells (monocytes and T-cells). In adults, suPAR serves as a prognostic biomarker after organ failures and neuronal injury and hypoxia [11], [12], [22]. High neonatal sera suPAR concentrations are reportedly associated with an increased risk of BPD, NEC, and LOS, which are all possible consequences of FIRS [23], [24], [25]. CB suPAR is considered to be of fetal origin because the large molecular size of suPAR makes it impossible to cross the placenta.Chorioamnionitis, sterile or microbial, is a strong stimulus to the preterm fetal immune system, and in adult patients, suPAR is reportedly a reliable marker for inflammation [17], [26]. Surprisingly, in our study population, no significant differences in CB suPAR were found between the study groups, and its predictability for HCA was poor. Interestingly, suPAR in AF was significantly higher in the HCA than in the non-HCA group. SuPAR is eliminated by kidneys, and AF is mostly fetal urine. AF suPAR reportedly has some potential in predicting HCA [13].

Corticosteroids have a strong inhibitory effect on inflammation. Betamethasone was administered to all patients after PPROM to promote fetal lung maturation. Studies on anti-inflammatory medication as a treatment, for instance in inflammatory bowel disease, cancer, and viral sepsis (COVID−19), demonstrated a significant decrease in suPAR levels after treatment [27], [28]. It is possible that this effect is also seen in CB suPAR.

Preterm neonates have small blood volumes and repeated blood draws can cause iatrogenic anemia and pain. CB could be useful to avoid those effects. Blood culture positivity is the golden standard in neonatal sepsis;however clinically suspected sepsis is 10 times more common [29], [30]. CB biomarkers can reflect the intrauterine status of the fetus. A combination of biomarkers would be more reliable for the diagnosis of FIRS and neonatal sepsis [31], [32], [33], [34].

## Limitations

The study had a small sample size.There were relative few cases of severe neonatal morbity to be evaluated.In addition, we lacked data on long-term morbidities, such as cerebral palsy. These limitations weakened the certainty of our results. Furthermore, it would have also been interesting to study repeated samples of neonates; for now, we only have samples right after birth.

## Strengths

This was a prospective study. We collected the AF and CB samples using the same protocol, and we were able to estimate biochemical markers in different compartments. All parturients received similar treatment with corticosteroids and antibiotics.

## Conclusion

In our study population, CB IL-6 had the best predictivity for HCA, although CB PCT also seems to be a promising biomarker. In terms of CB suPAR, the findings were insignificant. A larger study is needed to further investigate the association between biochemical markers and HCA after PPROM. To the best of our knowledge, there is no previously published data on CB suPAR and HCA.

## CRediT authorship contribution statement

**Kati Jalkanen:** Writing – original draft, Investigation, Funding acquisition, Formal analysis, Data curation, Conceptualization. **Janne Aittoniemi:** Writing – review & editing, Validation, Methodology, Investigation, Formal analysis. **Heini Flinck:** Writing – review & editing, Investigation, Formal analysis. **Outi Tammela:** Writing – review & editing, Investigation, Data curation, Conceptualization. **Anita Virtanen:** Writing – review & editing. **Kati Tihtonen:** Writing – review & editing, Supervision, Project administration, Methodology, Investigation, Formal analysis, Data curation, Conceptualization. **Sinikka Ampuja:** Writing – review & editing, Investigation, Formal analysis. **Heini Huhtala:** Writing – review & editing, Software, Methodology, Formal analysis, Data curation.

## Declaration of Competing Interest

The authors have no relevant financial or non- financial interests to disclose.

## Data Availability

All relevant data analyzed during this study are included in this published article and its supplementary information files.
